# Increased Occurrence of Cutaneous Leiomyomas and Dermatofibromas in Patients with Uterine Leiomyomas without Fumarate Hydratase Gene Mutations

**DOI:** 10.3390/dermatopathology10030032

**Published:** 2023-08-04

**Authors:** Elena Campione, Monia Di Prete, Gaetana Costanza, Andrea Saggini, Sara Agostinelli, Alessandro Terrinoni, Federica Centofanti, Maria Cristina Rapanotti, Luca Bianchi, Amedeo Ferlosio, Maria Giovanna Scioli, Augusto Orlandi

**Affiliations:** 1 Dermatology Unit, Department of Systems Medicine, University of Rome Tor Vergata, Via Montpellier 1, 00133 Rome, Italy; cmplne01@uniroma2.it (E.C.); luca.bianchi@uniroma2.it (L.B.); 2 Institute of Anatomic Pathology, Department of Biomedicine and Prevention, University of Rome Tor Vergata, Via Montpellier 1, 00133 Rome, Italy; diprete.monia@gmail.com (M.D.P.); costanza@med.uniroma2.it (G.C.); andreasaggini@gmail.com (A.S.); agostinelli.sara@hotmail.it (S.A.); federica.centofanti@gmail.com (F.C.); cristinarapanotti@yahoo.it (M.C.R.); ferlosio@med.uniroma2.it (A.F.); scioli@med.uniroma2.it (M.G.S.); 3 Department of Experimental Medicine and Biochemical Sciences, University of Rome Tor Vergata, 00133 Rome, Italy; alessandro.terrinoni@uniroma2.it; 4 Department of Biomedical Sciences, Catholic University Our Lady of Good Counsel, 1000 Tirana, Albania

**Keywords:** cutaneous leiomyoma, dermatofibroma, uterine leiomyoma, vitamin D, TGFβ-1

## Abstract

Leiomyomas are smooth muscle-derived benign neoplasms that can affect all organs, most frequently in the uterus. Fumarate hydratase gene (FH) mutation is characterised by an autosomal dominant disease with increased occurrence of renal tumours, but also by cutaneous (CLs) and uterine leiomyomas (ULs). So far, an increased occurrence of skin tumours in non-mutated patients with ULs has not been verified. To this aim, a case-group of women who were FH non-mutated patients surgically treated for ULs (*n* = 34) was compared with a control-group (*n* = 37) of consecutive age-matched healthy women. The occurrence of skin neoplasms, including CLs and dermatofibromas (DFs), was evaluated. Moreover, the microscopic features of FH non-mutated skin tumours were compared with those of an age-matched population group (*n* = 70) who presented, in their clinical history, only one type of skin tumour and no ULs. Immunohistochemical and in vitro studies analysed TGFβ and vitamin D receptor expression. FH non-mutated patients with ULs displayed a higher occurrence of CLs and DFs (*p* < 0.03 and *p* < 0.001), but not of other types of skin tumours. Immunohistochemistry revealed a lower vitamin D receptor (VDR) expression in CLs and DFs from the ULs group compared with those from the population group (*p* < 0.01), but a similar distribution of TGFβ-receptors and SMAD3. In vitro studies documented that TGFβ-1 treatment and vitamin D3 have opposite effects on α-SMA, TGFβR2 and VDR expression on dermal fibroblast and leiomyoma cell cultures. This unreported increased occurrence of CLs and DFs in FH non-mutated patients with symptomatic ULs with vitamin D deficiency suggests a potential pathogenetic role of vitamin D bioavailability also for CLs and DFs.

## 1. Introduction

Leiomyomas are benign smooth muscle-derived neoplasms which can affect all organs and tissues, most frequently in the uterus [1,2]. Leiomyomas consist microscopically of a benign proliferation of smooth muscle cells [2,3]. Uterine leiomyomas (ULs), also clinically known as fibroids, are the most frequent gynaecological tumours, affecting from 20% to 50% of reproductive-age females [4,5]. Genetic mutations and tumorigenesis of ULs are not completely identified [6]. Cutaneous leiomyomas (CLs) are far less common than ULs, although their incidence might be underestimated [7,8]. Clinically, CLs are skin-coloured or pink-brown, smooth, dome-shaped papules or nodules, of 0.2–2.0 cm in diameter [7]. CLs are subclassified into piloleiomyomas, angioleiomyomas and genital and nipple leiomyomas, based on differentiation towards the arrector pili muscle, the vascular muscular layer and the dartos and areolar smooth muscle, respectively [8,9]. Cutaneous dermatofibroma (DF) is another frequent benign mesenchymal dermal tumour. Clinically, DF generally appears as a hard, solitary, slow-growing, rounded papule with a variable colour, from brownish to tan [10]. DFs usually are composed of disordered collagen laid down by several cell types with histiocytic, myofibroblastic and fibroblastic features [11]. Although generally sporadic and single, familial predisposition in developing multiple leiomyomas has been reported along with the increased risk for renal cell carcinoma in an autosomal dominant pattern called hereditary leiomyomatosis and renal cell cancer syndrome (HLRCC) [12]. The latter depends on inactivating germline mutations of the fumarate hydratase (*FH*) gene, located on chromosome 1q42.3-43 [3,13]. CLs in association with multiple ULs, in the absence of *FH* germline mutations, have been clinically observed, but their frequency and that of other skin tumours not investigated [14]. The pathogenesis of sporadic leiomyomas and DFs is still far from being completely understood; genetic abnormalities, hormones, growth and angiogenic factors have been suggested [6,14,15]. Concerning the pathogenesis of sporadic ULs, mutations of mediator complex subunit 12 of exon 2 have been described [16]. Leiomyomatous cell proliferation and collagen deposition derive from a complex process involving multiple processes, including transforming growth factor β (TGFβ)- and vitamin D (VitD)-related pathways [17,18]. TGFβ is implicated in several cell processes, including development, proliferation, differentiation and migration, and stimulates extracellular matrix production, leading to fibrosis during the wound healing process [19,20]. Three isoforms of TGFβ (-1, -2 and -3) compete for the same receptors (TGFβ-RI and -RII), with different functions and tissue expression patterns [11,21]. In the adult life, TGFβ-1 is the most common and biologically relevant isoform and promotes, along with TGFβ-2, fibrosis during wound healing, whereas TGFβ-3 displays an anti-fibrotic role [22,23]. TGFβ-R signaling is generally regulated by the SMAD pathway, but an alternative SMAD-independent pathway has been also described [24,25]. Several studies showed that VitD and its receptor (VDR) display an antagonist role in the TGFβ pathway, in particular, in ULs [26]. In the present study, we aimed to investigate the frequency of CLs and DFs in *FH* non-mutated patients with a history of symptomatic ULs compared to a control group of age-matched *FH* non-mutated women without history or evidence of ULs. The potential pathogenetic role of VitD-TGFβ signalling was also investigated.

## 2. Materials and Methods

### 2.1. Patients and Clinical Investigation

The ULs group included 34 women (aged 30–69 years), referring to the Department of Obstetrics and Gynaecology of our University Hospital between January 2010 and July 2012, with symptomatic ULs, which were successively surgically treated (myomectomy or hysterectomy). The control group (*n* = 37) was obtained selecting age-matched (±5 years) consecutive women attending the Gynaecology Department for uterine clinical evaluation, which was unremarkable. Each patient underwent a clinical evaluation in the Department of Dermatology, and any suspect skin lesion removed and submitted for histologic examination. Exclusion criteria consisted in *FH* germline mutations (see after), VitD supplementation in the previous 3 months, abnormal Pap smear and primary or secondary immunodeficiency. Previous surgery for ULs was an exclusion criterion for control subjects. Data were collected via direct interview, clinical examination and review of medical records regarding pregnancy, uterine surgery, hormonal contraceptive use and menstruation pattern (period flow and symptoms). Body-mass index and smoking habits, history of hypertension, diabetes, coronary artery disease, recurring headache, cancer, familiarity for ULs or other gynaecological or renal neoplasms or any other type of cancer were also recorded. To evaluate the morbidity associated with ULs, women were asked about their age at diagnosis, ULs-associated symptoms and age at symptoms’ manifestation. Moreover, any history of infertility or recurrent pregnancy loss were further investigated. Patients with ULs underwent transvaginal and renal ultrasonography, as well as abdominal computed tomography scan. Complete blood count as well as serum testing for circulating 1,25(OH)2-D3 (VitD3), parathyroid hormone, calcitonin, phosphorus and calcium were also performed in all patients. VitD deficiency was defined for plasma values <20 ng/mL.

Afterwards, we selected a group of age-matched (±5 years) consecutive subjects (*n* = 70; population group), who presented in their history a microscopic diagnosis of only one sporadic UL (*n* = 30), CL (*n* = 10) and DF (*n* = 30), and tumour tissues were compared with the case-group. The study protocol was in accordance with the ethical guidelines of the Declaration of Helsinki and approved by the Institutional Ethics Board (n. 0013157/2015). All participants gave informed written consent.

### 2.2. FH Mutation Analysis

To exclude FH mutations, DNA extraction was performed from blood samples using the Wizard Genomic DNA Purification Kit (Promega Italia Srl, Milan, Italy). DNA was amplified by PCR using primers derived from genomic sequences within *FH* gene flanking each exon. PCR was performed by adding 100 ng of gDNA to a 50 µL reaction containing 0.2 µM of each primer, 200 µM dNTPs, 1.5 mM MgSO_4_ and 2.5 U of Platinum Taq DNA Polymerase High Fidelity (Invitrogen, Thermo Fisher Scientific, Waltham, MA, USA). A denaturing cycle of 95 °C for 4 min was followed by 40 cycles of 40 s at 94 °C, 40 s at 55 °C and 3 min at 72 °C. PCR product size and quality were checked on a 0.8% agarose gel, purified with the Qiaex Extraction Kit (Qiagen, Manchester, UK) and sequenced using amplification and additional internal primers.

### 2.3. Histological and Immunohistochemical Studies

Paraffin sections of formalin-fixed samples of uterine and skin neoplasms were stained with Haematoxylin–Eosin. The following histological parameters were assessed: hypercellularity, symplastic-type nuclear atypia and nucleolar prominence with perinucleolar clearing [27]. Immunohistochemistry was performed by deparaffinising, rehydrating and treating serial sections in sequence with 0.3% H_2_O_2_ [28] and then incubating with anti-TGFβ (1:50, Santa Cruz Biotecnology, Santa Cruz, CA, USA), anti-TGFβ-RI (1:200, Santa Cruz Biotecnology) and anti-TGFβ-RII (1:200, Santa Cruz Biotecnology) [29], a monoclonal rabbit anti-phosphorylated SMAD3 (1:400; S423 + S425, EP823Y; Abcam, Cambridge, UK), and mouse anti-VDR (1:150, clone D-6; Santa Cruz Biotechnology). All procedures were performed at room temperature using positive and negative controls; 3,3 diaminobenzidine was used as final chromogen. Stained sections were analysed independently and blindly reviewed by two of the authors, with an interobserver variability <5%. Positive internal controls in normal skin included dermal fibroblasts for TGFβ (as mild immunopositivity) and follicles and sebaceous glands for TGFβ-RI and TGFβ-RII (strong immunopositivity) [11,20]. Semiquantitative scoring of immunoreactivity was performed according to staining intensity (0: absent; 1: mild; 2: medium; 3: strong; 4: very strong) and percentage of positive cells (0: <5%; 1: 5–25%; 2: 25–50%; 3: 50–75%; 4: >75%). Percentage and intensity scores were summarised in a final score, ranging from 0 to 8 [11].

### 2.4. Cell Culture

Human dermal fibroblasts were isolated ex vivo from the dermal tissue surrounding a neck cyst to mimic the behaviour of DF cells. CL cells were obtained by enzymatic digestion of tumour biopsies from non-diagnostic tissues using 0.2% type I collagenase at 37 °C for 4 h (Policlinico Tor Vergata Ethics Committee n. 0013157/2015). Cells were cultured in Dulbecco’s modified Eagle’s medium (DMEM, Sigma Aldrich, Milan, Italy) supplemented with 15% fetal bovine serum and used between the 2nd and 5th passage. As concerning treatments, cells were kept in the same medium and incubated with 5 ng/mL TGFβ-1 and 10 nM 1.25(OH)_2_D3 (Sigma Aldrich), alone or in combination, for 2 days [30].

### 2.5. Western Blot Analysis

After isolation, content determination and electrophoresis, proteins were electroblotted [31] and incubated with a monoclonal rabbit anti-phosphorylated SMAD3 (1:1000; S423 + S425, EP823Y; Abcam), anti-SMAD3 (1:1000; EP568Y; Abcam), polyclonal rabbit anti-TGFβ-RI (1:200; Santa Cruz Biotechnology), anti-TGFβ-RII (1:200; Santa Cruz Biotechnology), mouse anti-VDR (1:100, D-6; Santa Cruz Biotechnology), anti-α-smooth muscle actin (α-SMA, 1:000 Dako Cytomatic, Glostrup, Denmark), anti-total tubulin and anti-GAPDH antibodies (Sigma Aldrich) following horseradish peroxidase conjugate goat anti-rabbit or anti-mouse IgGs (Pierce, Rockford, IL, USA). Specific complexes were revealed and quantified and densitometric blot analysis performed in three independent experiments [32].

### 2.6. Statistical Analysis

Patients were subdivided according to clinical and pathological features. Analysis of two-by-two contingency tables were performed to estimate the association between site-specific and presence or absence of skin disorder and other possible risk factors. Pearson’s Chi-square test and crude ORs with their confidence intervals (95% CI) were estimated using the first category as the reference group. Significance of OR can be noticed from its CI. Continuous variables were categorised according to biological considerations or conventional cut-off points. Immunohistochemical and in vitro results were analysed as arithmetic mean ± standard error of the mean (SEM). Data were analysed by one-way analysis of variance (ANOVA) followed by a Bonferroni post hoc test and using the Student *t*-test. Differences were considered statistically significant for *p* < 0.05. All statistical analyses were performed with SSPS V20 (Stat Corp, College Station, TX, USA) [33].

## 3. Results

### 3.1. Clinical and Serological Data

Demographic, clinical-pathological and serological data are summarised in Table 1 and Table 2. As indicated in Table 2, ULs patients had increased clinical evidence of anaemia (defined as Hb < 12 g/dL; OR 2.99, *p* < 0.02), menometrorrhagia (OR 5.70, *p* < 0.001) and headaches (OR 3.07, *p* < 0.02). Patients with symptomatic ULs also showed a higher frequency of serum VitD3 deficiency compared to the control group (*p* < 0.001). There were no significant differences between ULs and control patients regarding other gynaecological features, such as period flow, parity, mode of delivery, infertility and recurrent pregnancy loss.

### 3.2. Genomic Analysis and Frequency of Cutaneous Tumours

Analysis of the exon-specific sequence, spanning all the coding regions of the *FH* gene, did not show any pathologic mutations in the enrolled patients. As reported in Table 3, after a dermatological examination and successive surgical excision, six single CLs were diagnosed in the ULs group (20.6%), whereas there was only one in the control group (2.7%). Additionally, DFs were diagnosed in 15 ULs patients (44.1%) and only in 3 patients in the control group (8.1%). In one ULs case (2.9%), the association of CL and DF was documented. Statistical analysis documented that the occurrence of CLs and DFs in ULs patients was significantly greater (OR 7.71, *p* < 0.03 for CLs; OR 7.93, *p* < 0.001 for DFs). By contrast, no difference in the occurrence of melanocytic nevi or other epidermal tumours was observed (Table 3).

### 3.3. Microscopic and Immunohistochemical Analysis of the Dermis

**Figure 1:**
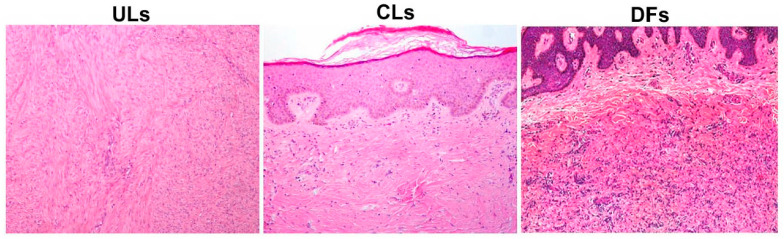
Microscopic features of uterine (UL) and cutaneous leiomyomas (CL) and dermatofibroma (DF) (Haematoxylin–Eosin staining, original magnification: 100×). ULs group and the population group revealed similar and typical histological features. ULs and DFs consisted of a proliferation of benign-appearing smooth muscle cells along with haphazard, collagen-rich extracellular matrix deposition. All DFs were typically characterised by collagen and proliferation of fibrohistiocytic cell accumulation, with overlying hyperpigmented keratotic epidermis. No differences in cellularity, symplastic-type nuclear atypia or prominent nucleoli were observed.

A microscopic and immunohistochemical evaluation of ULs, CLs and DFs from the ULs group and the population group revealed similar and typical histological features. ULs and DFs consisted of a proliferation of benign-appearing smooth muscle cells along with a haphazard, collagen-rich extracellular matrix deposition. All DFs were typically characterised by collagen and the proliferation of fibrohistiocytic cell accumulation, with an overlying hyperpigmented keratotic epidermis (Figure 1).

**Figure 2:**
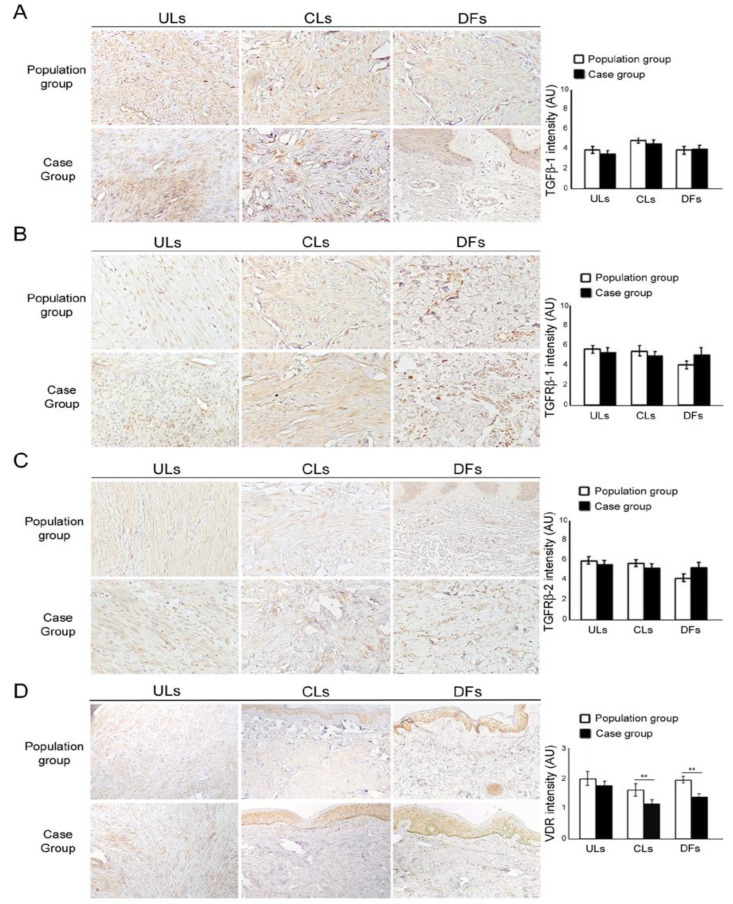
Evaluation of TGFβ-1, TGFβ-RI and TGFβ-RII expression in population (white) and uterine leiomyomas (ULs) patients (black). (A-B-C) From the top to the bottom, TGFβ-1, TGFβ-RI and TGFβ-RII immunostainings in matching lesions (ULs), cutaneous leiomyomas (CLs) and dermatofibromas (DFs) in normal population and ULs groups, respectively, with no statistical differences. (**A**) TGFβ-1, (**B**) TGFβ-RI and (**C**) TGFβ-RII stains. Original magnification: 100×. (**D**) VitD receptor (VDR) expression in population (white) and uterine leiomyomas (ULs; black) groups. (**A**) VDR immunohistochemical expression is different in the histotypes analysed (VDR stain. Original magnification: 100×). (**B**) CLs and DFs showed a lower VDR expression in ULs group compared to the population group, as confirmed by statistical analysis (** *p* < 0.01).

No differences in cellularity, symplastic-type nuclear atypia or prominent nucleoli were observed. Semiquantitative analysis did not reveal any significant difference in the immunohistochemistry for TGFβ-1, TGFβ-RI and TGFβ-RII expression among the ULs, CLs and DFs from the groups (Figure 2A–C). Instead, VDR expression was lower in the CLs and DFs from the case group compared to those from the control population (Figure 2D, *p* < 0.01).

**Figure 3:**
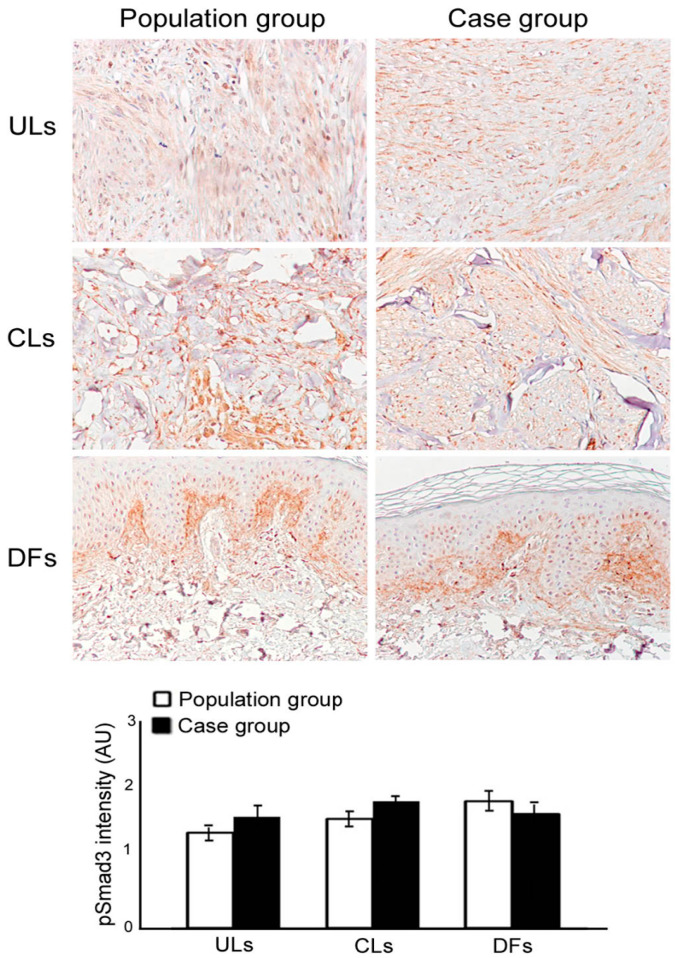
Phosphorylated SMAD3 expression in general population (white) and uterine leiomyomas (ULs; black) groups. Phosphor-SMAD3 shows a moderate expression in the analysed lesions (uterine leiomyomas (ULs), cutaneous leiomyomas (CLs) and dermatofibromas (DFs) without statistically significant differences between groups. SMAD3 stain (original magnification: 100×).

Finally, a semiquantitative evaluation of phosphorylated SMAD3 expression did not reveal any significant difference among the groups (Figure 3).

### 3.4. 1,25(OH)_2_D3 and TGFβ-1 Have Opposite Effects on VDR, TGFβ-RII and α-SMA Expression In Vitro

**Figure 4:**
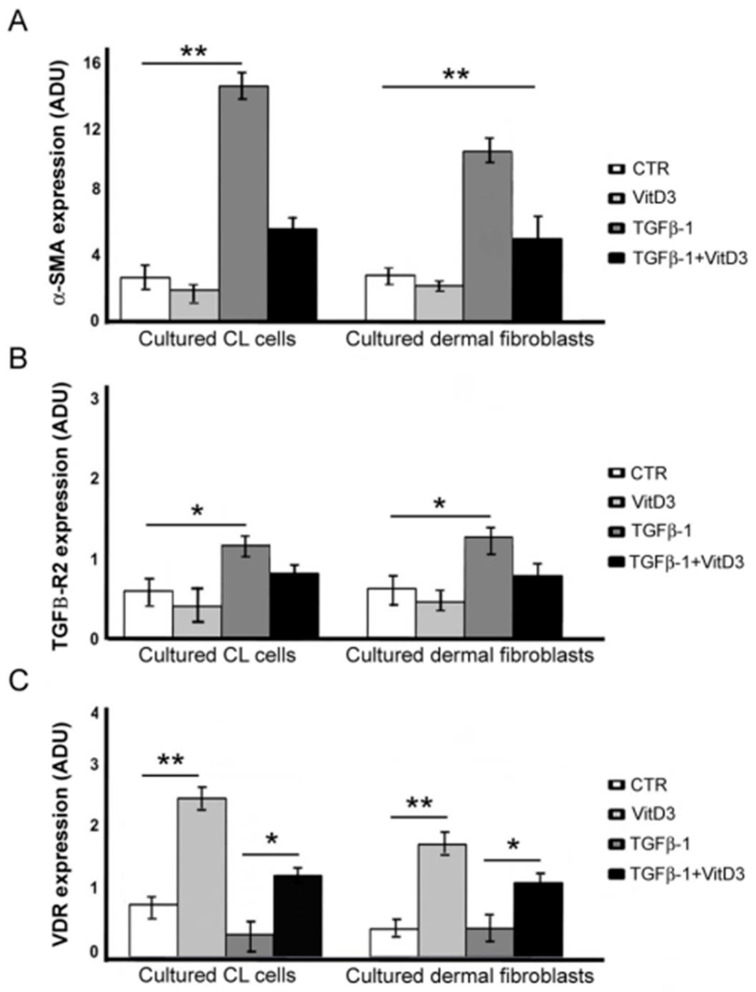
Influence of TGFβ-1 and vitamin D (VitD) on α-alpha smooth muscle actin (SMA), TGFβ-RII and vitamin D receptor (VDR) expression in cutaneous leiomyoma (CLs) cells and dermal fibroblasts. (**A**) Blot analysis showing the expression of α-SMA increases after TGFβ-1 treatment in both CL cells and dermal fibroblasts (** ANOVA; *p* < 0.01). The effect is prevented by VitD treatment. (**B**) Blot analysis showing TGFβ-RII expression increases after TGFβ-1 treatment (* ANOVA; *p* < 0.001) and decreases by adding VitD in both CL cells and dermal fibroblasts. (**C**) VDR expression increases after VitD and decreases after TGFβ-1 treatment (** ANOVA; *p* < 0.01) in CL cells and dermal fibroblast cultures.

In order to highlight a possible alteration of VDR, phosphorylated SMAD3, TGFβ-RI and TGFβ-RII expression in a fibrogenetic growth of CL and DF cells, human dermal fibroblasts and CL cells were cultured and treated with TGFβ-1 and VitD. As reported in Figure 4, blot analysis showed increased α-SMA levels after TGFβ-1 treatment compared to the serum control (ANOVA; *p* < 0.01). Instead, the adding of 1,25(OH)2D3 prevented the TGFβ-1-induced increase of α-SMA in both CL cells and dermal fibroblast cultures (ANOVA; *p* < 0.01). Moreover, TGFβ-1 treatment induced the increase of TGFβ-RII expression (ANOVA; *p* < 0.05), but not of TGFβ-RI. The addition of VitD counteracted the TGFβ-RII upregulation. VDR expression increased after adding VitD (ANOVA; *p* < 0.01), which was prevented by the combined use of TGFβ-1 in both CL cells and dermal fibroblast cultures. No difference was found in phosphorylated SMAD3 expression.

## 4. Discussion

In this study, we reported an increased occurrence of CLs and DFs in *FH* wild-type patients suffering from symptomatic ULs compared to age-matched women without a history of ULs. In addition, our data suggest a previously unreported association between the occurrence of CLs or DFs in FH wild-type patients with symptomatic ULs [34,35]. No increased frequency of other melanocytic or non-melanocytic skin tumours occurred among the FH non-mutated females of the ULs group compared to the control group, suggesting a specific link in the occurrence of benign mesenchymal tumours in multiple organs.

We also documented that patients of the ULs group had lower levels of serum circulating VitD compared to the control group, supporting a potential link between a VitD deficiency and the occurrence of symptomatic ULs [36,37]. In fact, epidemiologic data as well as in vitro and in vivo experimental studies provided robust evidence supporting an antagonistic role of 1α,25(OH)2D3, the active form of VitD, in modulating UL growth [18,36,37]. We also observed an association between low levels of serum 1α,25(OH)_2_D3 and an increased risk of UL [26,37]. Additionally, the administration of VitD analogues has been proposed as a potential option in the treatment of ULs [18]. Our data seem to support a potential role of VitD deficiency also for the occurrence of benign cutaneous mesenchymal tumours. Similar to other lipid-soluble steroid hormones, 1α,25(OH)2D3 acts through a classic nuclear as well as a rapid non-genomic pathway [38]. In the classic nuclear pathway, VitD activates VDR nuclear receptors, which bind specific VitD-binding response elements in the promoter/enhancer region of target genes, modulating gene expression in a cell- and tissue-specific manner [18,39]. In the rapid non-genomic pathway, active VitD binds to membrane-bound VDR receptors, activating several pathways, including those involving MEK-ERK, PLCγ-PKC and cAMP-PKA signalling [38]. VitD has been found to play a tumour-suppressor role through the inhibition of cellular proliferation and angiogenesis [38,40,41]. Additionally, VitD has been documented as an anti-fibrotic agent in both non-neoplastic chronic diseases and in tumour progression experimental models [25,42]. The latter indicated that VitD treatment decreased the expression of PCNA, CDK1, BCL-2 and COMT [30,40,41,43].

There is growing evidence supporting a role of aberrant TGFβ/SMAD signalling in the development and growth of ULs [44,45]. TGFβ is typically regarded as a master regulator of fibrotic processes and its three isoforms are capable of exerting different biological functions in a highly cell type-specific and tissue-dependent manner [44]. TGFβ ligands most commonly form heterotetrameric complexes with TGFβ-RI and -RII, thus propagating signalling to the receptor-activated SMADs (R-SMADs) [46,47]. Phosphorylated R-SMADs interact with the common mediator SMAD4 to form oligomeric complexes that translocate to the nucleus and work as bona fide transcription factors [46]. The key role of the TGFβ/SMAD pathway in fibrogenesis was confirmed in non-neoplastic conditions, cancer-associated fibroblasts [38,42] and in the pathogenesis of both leiomyomas and DFs [1,11]. An increased transcription and expression of TGFβ-3 was found in ULs compared to the normal myometrium [48,49]. We did not find any significant difference in the immunohistochemical expression of TGFβ-RI and TGFβ-RII among the ULs patients and the population control. In addition, microscopically, there was a substantial overlapping of the tissue lesions of the two groups. UL cells seemed refractory to the antiproliferative effects of TGFβ-3, whereas TGFβ signalling induced the expression of profibrotic proteins, such as type-I and -III collagen [1]. It has been reported that there is an overexpression of SMAD3 and their phosphorylated isoforms in UL compared to normal myometrium [50]. In our cohort of patients, phosphorylated SMAD3 did not vary when comparing CLs and DFs from the UL group with those from the population group, while VDR expression was lower than in the cases compared to the population subjects. Active VDR apparently interferes with TGFβ signalling through both genomic (i.e., competition with SMAD-4-dependent transcription complexes) and non-genomic (i.e., inhibition of R-SMADs phosphorylation) mechanisms, resulting in a down-regulation of TGFβ-induced proteins [40,41,42,43]. Based on the literature data reporting the proliferative and pro-fibrotic effect of TGFβ-1 treatment and the antagonistic activity of VitD3 on fibroblasts [38,40,51], we tested the hypothesis of a role of VitD in CL and DF pathogenesis and an antagonistic or synergic relationship with TGFβ. We treated CL cells and dermal fibroblasts with VitD and TGFβ-1 and analysed the subsequent expression of different proteins involved in the VitD/TGFβ-1 pathway and fibrotic activity. In vitro experiments seem to support the antagonist role between VitD and TGFβ-1, modulated by VDR, TGFβ-RII and α-SMA.

In conclusion, we have described a previously unreported increased occurrence of CLs and DFs in FH non-mutated females with ULs compared with the control population. No increased frequency of other melanocytic or non-melanocytic skin neoplasms occurred. This is suggestive to hypothesise that a VitD deficiency, facilitating TGFβ signalling transduction, might result in compensatory decreases in TGFβ autocrine and paracrine secretion by neoplastic cells and the tumour environment through an inhibitory feedback loop [46]. Although suggestive, the hypothesis of the role of VitD in the pathogenesis of CL and DF needs further studies to be confirmed.

## Figures and Tables

**Table 1:** Demographic and clinical-pathological data in uterine leiomyomas (ULs; *n* = 34) and control (*n* = 37) groups.

Variable		*n* = 71	%
Age (Mean ± SEM)		46.5 (±16.5 years)	
Body-mass index	Underweight	19	26.76
	Normal weight	38	53.52
	Overweight	14	19.72
Smoking (>10 cigarettes/day)	no	30	42.25
	yes	41	57.75
Parity	Nulliparous	31	43.66
	Parous	40	56.34
Mode of Delivery	Spontaneous	20	28.17
	Operative	2	2.82
	Caesarean section	18	25.35
	Nulliparous	31	43.66
Infertility	no	51	71.83
	yes	20	28.17
Period flow	Regular period	19	26.76
	Irregular period	24	33.80
	Menopause	18	25.35
	Contraceptive use	10	14.08
Uterine leiomyomas	no	37	52.11
	yes	34	47.89
Cutaneous leiomyomas	no	63	88.73
	yes	8	11.27
Cutaneous dermatofibromas	no	44	61.97
	yes	27	38.03

**Table 2:** Anamnestic data, clinical symptoms and serological parameters in uterine leiomyomas (ULs; *n* = 34) and control (*n* = 37) groups.

	ULs Group	Control Group	OR	95% CI
	(*n* = 34)	(*n* = 37)		
Anaemia (<12 g/dL)				
no	15 (44.0)	26 (71.0)	-	-
yes	19 (56.0)	11 (29.0)	2.99 (*p* < 0.02)	1.13–7.96
Menometrorrhagia				
no	12 (35.2)	28 (75.7)	-	-
yes	22 (64.7)	9 (24.3)	5.70 (*p* < 0.0006)	2.04–15.96
Headache				
no	11 (32.4)	22 (59.6)	-	-
yes	23 (67.6)	15 (40.5)	3.07 (*p* < 0.02)	1.16–8.12
Vitamin D level (ng/mL)				
<10	20 (58.8)	1 (2.7)	-	-
11–20	7 (20.6)	14 (37.8)	40 (*p* < 0.001)	4.42–362.39
21–50	7 (20.6)	22 (59.5)	62.9 (*p* < 0.001)	7.10–556.69
Family History of UL				
no	12 (35.3)	22 (59.6)	-	-
yes	22 (64.7)	15 (40.5)	2.69 (*p* < 0.04)	1.03–7.04

**Table 3:** Frequency of nevi, cutaneous leiomyomas (CLs), dermatofibromas (DFs) and other neoplasms among uterine leiomyomas (ULs; *n* = 34) and control (*n* = 37) groups.

	ULs Group (*n* = 34)	Control Group (*n* = 37)	OR	95%CI
CLs				
no	27 (79.4)	36 (97.3)	-	-
yes	7 (20.6)	1 (2.7)	0.13 (*p* < 0.03)	0.11–1.14
DFs				
no	19 (55.9)	34 (91.9)	-	-
yes	15 (44.1)	3 (8.1)	7.93 (*p* < 0.001)	2.02–31.03
Nevi				
no	18 (52.9)	19 (51.4)	-	-
yes	16 (47.1)	18 (48.6)	0.94	0.37–2.38
Other				
no	19 (55.9)	18 (48.6)	-	-
yes	15 (44.1)	19 (51.4)	0.75	0.29–1.9

## Data Availability

Data are available on reasonable request. The datasets generated and/or analysed during this study are available from the corresponding author on reasonable request.
